# Basal ganglia neurophysiological markers of non-motor symptoms in Parkinson's disease: A systematic review

**DOI:** 10.1177/1877718X261459224

**Published:** 2026-06-23

**Authors:** Bart J Keulen, Martijn Beudel, Arjan Malekzadeh, Rob MA de Bie, Bart EKS Swinnen

**Affiliations:** 1 Department of Neurology, Amsterdam UMC, Amsterdam Neuroscience, Amsterdam, The Netherlands; 2 Medical Library, Amsterdam UMC, Amsterdam, The Netherlands; 3 Department of Neurology, University Hospitals Leuven, Leuven, Belgium

**Keywords:** Parkinson's disease, non-motor symptoms, deep brain stimulation, local field potentials, subthalamic nucleus, globus pallidus interna

## Abstract

**Background:**

Deep brain stimulation (DBS) for Parkinson's disease (PD) primarily improves motor symptoms but leaves non-motor symptoms (NMS) largely unattended. Neurophysiological markers associated with specific symptoms could improve DBS programming. We systematically reviewed the evidence linking basal ganglia local field potentials (LFP) to NMS in PD.

**Methods:**

The literature search (Medline, Embase, Scopus, and Web of Science) on August 20, 2024 yielded 1066 records. Studies were included if they focused on patients with idiopathic PD treated with DBS of the subthalamic nucleus (STN) or globus pallidus interna (GPi) and reported on the relationship between LFP data and NMS. The study risk of bias was evaluated using the Prediction Model Risk of Bias Assessment Tool (PROBAST). A narrative synthesis of results was provided.

**Results:**

Twenty-one studies were included, focusing on impulse control disorders (n = 8), sleep-wake disorders (n = 5), depressive symptoms (n = 4), cognitive dysfunction (n = 3), hypomania (n = 1) and lower urinary tract symptoms (n = 1). Seven studies had a high risk of bias. Theta and alpha power in the STN were frequently associated with neuropsychiatric symptoms and cognitive function. Beta power in the STN and GPi was linked to sleep-wake disorders and urinary dysfunction.

**Conclusions:**

Overall, evidence on basal ganglia physiomarkers of NMS in PD remains limited. Further research is essential to develop patient-specific stimulation paradigms targeting NMS, which could significantly improve the quality of life of individuals with PD.

**Other:**

This systematic review was registered with the International Prospective Register of Systematic Reviews (CRD42024495284). There was no specific funding for this study.

## Plain language summary

Brain activity markers of non-motor symptoms in Parkinson's disease: a review of the scientific literature

Parkinson's disease patients often suffer from slowness of movement, muscle stiffness and tremor. One form of treatment is deep brain stimulation. During deep brain stimulation, brain tissue is electrically stimulated using electrodes implanted in the brain. This treatment improves motor symptoms but often not non-motor symptoms such as cognitive problems or mood disorders. With the use of markers which are linked to symptoms, deep brain stimulation treatment could be improved. Such markers can be found in brain activity measured around the electrodes. Much progress has been made in finding such markers of motor symptoms. However, this is less so for non-motor symptoms. A complete overview of the evidence on this topic does not exist. This is why we created an overview of the scientific literature on brain activity markers of non-motor symptoms in Parkinson's disease.

Our online search of the scientific literature resulted in 1066 unique studies. Studies were used in our review if they were on the subject of patients with Parkinson's disease treated with deep brain stimulation. Next to that, studies had to report on the relation between brain activity and non-motor symptoms. Twenty-one studies were used in our review, on the topics of impulse control disorders (8 studies), sleep-wake disorders (5 studies), depressive symptoms (4 studies), cognitive problems (3 studies), hypomania (1 study) and urinary symptoms (1 study). Two markers were linked to impulse control disorders, depressive symptoms, hypomania and cognitive problems. One marker was linked to sleep-wake disorders and urinary symptoms. These results show that brain activity markers of non-motor symptoms exist, but that the amount of evidence is limited. More research is needed to develop new treatment methods for non-motor symptoms. Such new treatment methods could improve the lives of patients with Parkinson's disease.

## Introduction

Parkinson's disease (PD) is a neurodegenerative disorder characterized by motor symptoms such as bradykinesia, rigidity, and tremor^1^ However, most patients also experience non-motor symptoms (NMS), including cognitive impairment, mood disturbances, sleep-wake disorders, and autonomic dysfunction.^2–4^ NMS are often not reported to healthcare providers and have a high impact on the quality of life of patients.^5,6^ While specific treatments are available for some NMS, high-quality evidence is limited, and their effectiveness remains suboptimal.^4,7^ This underscores the need for more targeted therapies addressing non-motor symptoms in patients with PD.

Deep brain stimulation (DBS) is an effective treatment for medication-refractory motor symptoms in PD.^8^ DBS has been associated with both improvements (e.g., depression and autonomic dysfunction) and deteriorations (e.g., verbal fluency and apathy) of several NMS. However, some of these changes may result from postoperative reductions in dopaminergic medications or from the surgery itself.^9–14^ DBS has advanced our understanding of the neurophysiology underlying PD by enabling subcortical local field potential (LFP) recordings from the target nuclei using the implanted DBS electrodes.^15,16^ These LFP signals represent the summed and synchronized activity of surrounding neural tissue and have been shown to harbor neurophysiological markers, or physiomarkers, indexing the severity of motor symptoms such as bradykinesia, rigidity, and dyskinesia.^17–19^ These physiomarkers hold promise for optimizing DBS programming^20,21^ and have led to the development of closed-loop or adaptive DBS.^22,23^

Physiomarkers indexing NMS would provide objective measures of symptom presence and severity and could guide DBS stimulation paradigms to improve the effectiveness of DBS. However, research in this area remains limited, and a comprehensive overview of existing studies is lacking. A recent study reviewed the literature on neurophysiology underlying mood, motivation, and behavioral symptoms in PD^24^ However, they did not systematically address the full spectrum of NMS. Instead, it primarily explored non-motor aspects through behavioral paradigms, regardless of whether NMS were present. To address these gaps, we aimed to systematically review the literature examining the relationship between basal ganglia LFPs and both the presence and severity of NMS in patients with PD.

## Methods

This systematic review was conducted and reported in compliance with the Preferred Reporting Items for Systematic Reviews and Meta-Analyses (PRISMA) guidelines.^25^ It was registered with the International Prospective Register of Systematic Reviews (PROSPERO) on December 21, 2023 (registration number: CRD42024495284).

### Search strategy

A comprehensive search was conducted in the following databases: MEDLINE (Ovid), Embase (Ovid), Scopus, and Web of Science, from inception to December 8, 2023, in collaboration with a medical information specialist (AM). The search was repeated on August 20, 2024. The search strategy included both controlled terms and free text terms for synonyms of ‘Parkinson's disease’ combined with synonyms of ‘local field potentials’ and both synonyms and specific types of ‘mood disorders’, ‘cognition disorders’, ‘autonomous disorders’ and other NMS of PD. The search was conducted without restrictions on date or language. Detailed search strategies for each database are available in the Supplementary Materials. Duplicate articles were removed using an in-house made deduplication tool.

### Selection process

The inclusion criteria for studies were: (1) studies involving patients with idiopathic PD, (2) patients treated with unilateral or bilateral DBS of the subthalamic nucleus (STN) or globus pallidus interna (GPi), and (3) studies examining the relationship between measures derived from basal ganglia LFP recordings and NMS of PD as measured through clinical assessments or tasks evaluating non-motor functions. LFP-derived measures could include the power or amplitude envelope of the LFP signal within a certain frequency range, or measures of the relationship between the LFP signal and other neurophysiological measures such as EEG. No restrictions were set on the type of physiomarker as long as it was derived from basal ganglia LFP signals recorded using DBS electrodes. Studies using tasks assessing non-motor functions were included only if the task was directly related to a specific NMS or if the task results were related to the presence or severity of NMS. Exclusion criteria included studies involving non-human subjects, editorials, narrative reviews, conference presentations, case reports, preprints, and non-peer reviewed studies.

Two researchers (BJK and BEKSS) independently screened all reports for eligibility based on their titles and abstracts. Disagreements were resolved through collective assessment of the full text. Additionally, the reference lists of all included articles were reviewed to identify further eligible studies. The selection process was managed using Rayyan (http://rayyan.qcri.org).^26^

### Data collection and analyses

The data were collected by a single researcher (BJK) using a standardized data extraction form. The following information was collected: patient demographics; number of participants in each group; number of hemispheres with LFP recordings; neuroanatomical location of LFP recordings; duration and setting of LFP recordings (e.g., intraoperative, perioperative, or at home); medication and stimulation status during LFP recording; LFP features; NMS assessment method; and the methods and outcomes of assessment of the relationship between LFP data and NMS.

All findings were grouped by LFP recording site and by NMS. A narrative synthesis was provided with results presented in both tables and text. Given the scarcity of studies addressing each NMS and the pronounced variability in study designs, methods, and outcomes, data heterogeneity and publication bias analyses were not feasible. Consequently, assessing the overall strength of the body of evidence was not considered useful and was not performed for this review.

The Prediction Model Risk of Bias Assessment Tool (PROBAST) was used to evaluate each included study for risk of bias and its applicability to the review question.^27,28^ PROBAST categorizes both risk of bias and concerns regarding applicability as “low”, “high”, or “unclear” across four domains. Two researchers (BJK and BEKSS) independently performed the risk of bias and applicability assessment. If their evaluations of an item differed by only one grade (e.g., “low” and “unclear” or “unclear” and “high”), the higher rating was applied. For items assessed as “low” by one researcher and “high” by the other, the researchers resolved discrepancies through discussion to reach consensus.

## Results

### Study selection and characteristics

**Figure 1.**
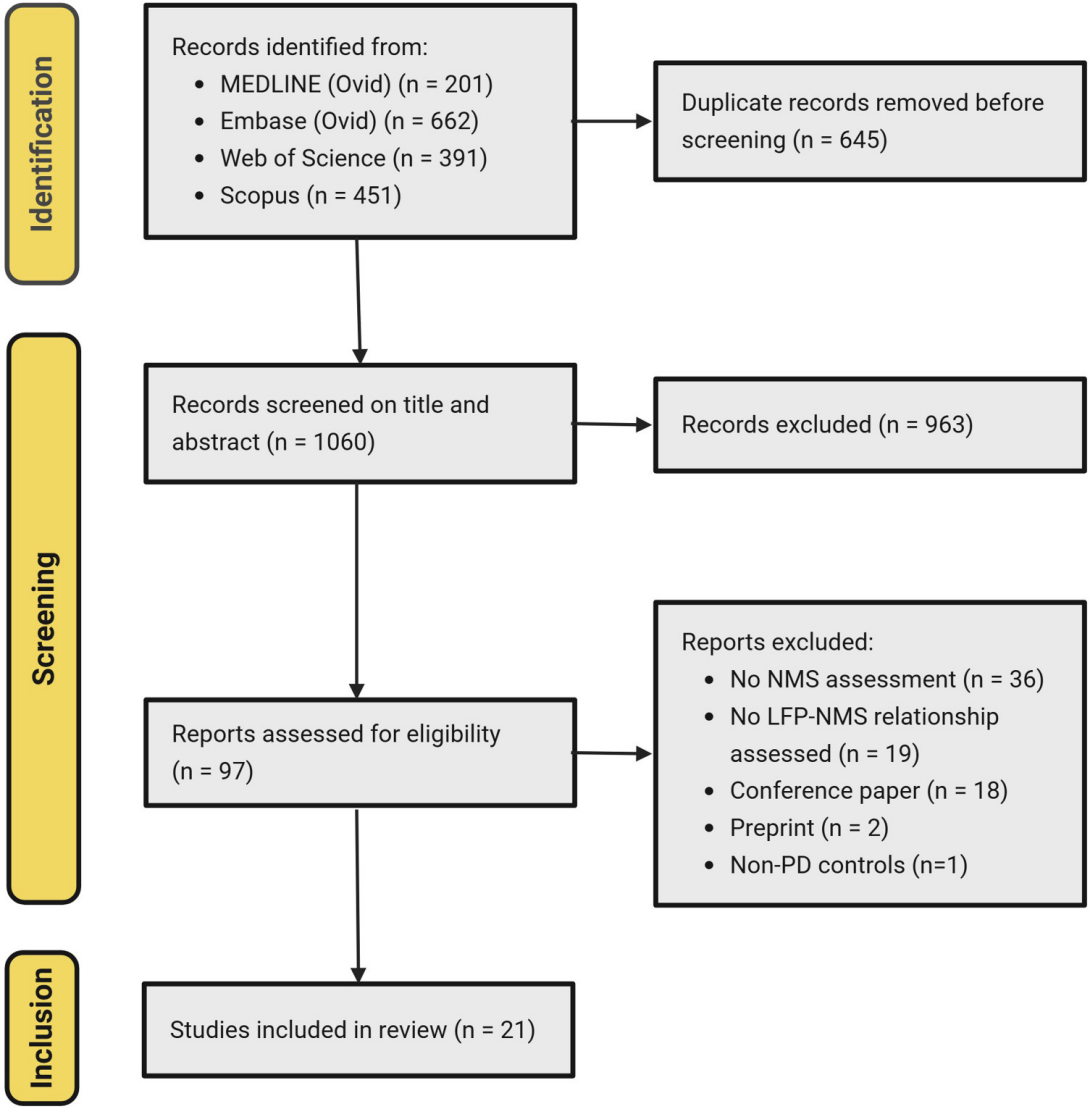
PRISMA flow diagram of the selection procedure. LFP: local field potentials; NMS: non-motor symptoms; PD: Parkinson disease.

The search strategy yielded 1066 unique records. Following title and abstract screening, as well as full-text review, 21 studies met the inclusion criteria (Figure 1). The NMS assessed in these studies included impulse control disorders (ICD; n = 8), sleep-wake disorders (n = 5), depressive symptoms (n = 4), hypomania (n = 1), cognitive dysfunction (n = 3) and lower urinary tract symptoms (LUTS; n = 1). Among the studies focusing on sleep-wake disorders, specific topics included sleep quality (n = 3), REM sleep behavior disorder (RBD; n = 3), and sleep fragmentation (n = 1). The studies recorded LFPs from the STN (n = 16), GPi (n = 2), or both (n = 3). The median number of study participants with NMS assessments (excluding controls) was 12 (range: 3–99). Detailed study characteristics are summarized in Table 1. Based on the PROBAST assessment, seven of the 21 studies were rated as having a “high” risk of bias, while six were rated “unclear” (Supplementary Table S1). Concerns regarding applicability were assessed as “high” for three studies and “unclear” for ten studies.

**Table 1.** Characteristics of the included studies for the subthalamic nucleus and globus pallidus interna, per non-motor symptom.

Subthalamic nucleus							
First Author	Year	Patients	Controls	NMS assessment	Task	DBS status	Med status
*Impulse control disorders*							
Alegre^29^	2013	4 (8)	6 (12)	Criteria by Weintraub^30^	Stop signal	Externalized	OFF, ON
Eisinger^31^	2022	15 (18)		QUIP-RS	Go/NoGo	Intraoperative, awake	OFF
Fumagalli^32^	2011	6 (12)	10 (20)	Unclear	Visual sentence verification	Externalized	ON
Manssuer^33^	2021	9 (18)	8 (16)	QUIP	Monetary incentive	Externalized	ON
Mazzoni^34^	2018	6 (12)	6 (12)	Psychiatric interview and SOGS	Economic decision-making	Externalized	ON
Ricciardi^35^	2021	6 (12)	17 (34)	QUIP-RS	None	Externalized	ON
Rodriguez-Oroz^36^	2011	10 (19)	18 (33)	Psychiatric evaluation	None	Externalized	OFF, ON
Rosa^37^	2013	8 (16)	9 (18)	Clinical interview and SOGS	Economic decision-making	Externalized	ON
*Depressive symptoms*							
Huebl^38^	2011	12 (24)		BDI	Emotional processing	Externalized	ON
Huebl^39^	2014	3 (6)	12 (23)	BDI	Emotional processing	Externalized	ON
Rappel^40^	2020	41 (N/A)		BDI	None	Intraoperative, awake	OFF
Sun^41^	2021	11 (20)	10 (20)	HDRS-17	None, anesthetized	Intraoperative, general anesthesia	OFF
*Hypomania*							
Chen^42^	2021	22 (24)	42 (80)	DSM-5 criteria	None	Intraoperative, awake	OFF
*Cognitive dysfunction*							
Anzak^43^	2011	8 (16)		Semantic and phonemic verbal fluency tasks		Externalized	ON
Rappel^40^	2020	96–99 (N/A)		FAB (n = 96) and ACE (n = 99)	None	Intraoperative, awake	OFF
Wojtecki^44^	2016	16 (28)		Semantic verbal fluency task		Externalized	ON
*Sleep-wake disorders*							
Hackius^45^	2016	4 (8)		Video-PSG	None	Externalized	OFF
Yin^46^	2024	17 (34)		Video-PSG and RBD-SQ	None	Externalized	OFF
Zhang^47^	2024	3 (6)		PSG: SFI and ARI	None	Chronic	OFF
*Lower urinary tract symptoms*							
Roy^48^	2018	5 (8)		ICIQ	Urinary voiding	Externalized	ON

Globus pallidus interna							
First Author	Year	Patients	Controls	NMS assessment	Task	DBS status	Med status
*Impulse control disorders*							
Eisinger^31^	2022	24 (28)		QUIP-RS	Go/NoGo	Intraoperative	ON
*Sleep-wake disorders*							
Verma^49^	2022	4 (8)		Video-PSG	None	Externalized	OFF
Yin^50^	2023	12 (24)		RBD-SQ and PSQI	None	Externalized	OFF
Yin^46^	2024	11 (22)		Video-PSG and RBD-SQ	None	Externalized	OFF
*Lower urinary tract symptoms*							
Roy^48^	2018	5 (9)		ICIQ	Urinary voiding	Externalized	ON

Number of patients and controls are denoted as: number of participants (number of hemispheres). ACE: Addenbrooke's Cognitive Examination; ARI: arousal index; BDI: Beck Depression Inventory; DBS: deep brain stimulation; DSM-5: Diagnostic and Statistical Manual of Mental Disorders, Fifth Edition; FAB: Frontal Assessment Battery; HDRS-17: 17-item Hamilton Depression Rating Scale; ICIQ: International Consultation on Incontinence Questionnaire; NMS: non-motor symptom; OFF: off levodopa medication state; ON: on levodopa medication state; PSG: polysomnography; PSQI: Pittsburgh Sleep Quality Index; QUIP: Questionnaire for Impulsive-Compulsive Disorders in Parkinson's Disease; QUIP-RS: Questionnaire for Impulsive-Compulsive Disorders in Parkinson's Disease – Rating Scale; RBD-SQ: REM Sleep Behavior Disorder Screening Questionnaire; SFI: sleep fragmentation index; SOGS: South Oaks Gambling Screen.

### Outcomes

An overview of the study findings are provided in Table 2. In most studies, the LFP feature used to correlate with NMS focused on the power of one or more frequency bands. The power of these bands was calculated over the entire recording,^35,36,41–44^ per event,^29,32–34,37–39,45,48,49^ or per sleep stage.^46,47,50^ Additional features included event-related potentials (ERP),^31^ subthalamic-cortical coherence,^29^ and subthalamic-EMG connectivity.^47^

**Table 2.** Summary of study results for the subthalamic nucleus and globus pallidus interna, per non-motor symptom and frequency band.

Frequency band	Results
**Subthalamic nucleus**	
*Impulse control disorders*	
Delta	*Monetary incentive task:* negative correlation between QUIP scores and delta power during reward outcome^33^
Theta	*Resting-state:* higher subthalamic-cortical coherence in the group with ICD^36^
	*Monetary incentive task:* positively correlation between QUIP scores and theta power during reward outcome^29^
	*Stop signal task:* no association found^33^
Theta-alpha	*Resting-state:* higher relative increase from OFF to ON medication state in the group with ICD^36^
Alpha	*Resting-state:* correlation with BIS-11 scores but not QUIP-RS scores^35^
Low frequency	*Economic decision-making task*: lower preceding low-risk choices compared to high-risk choices during outcome presentation in patients with pathological gambling^34^
	*Economic decision-making task:* higher preceding low-risk choices compared to high-risk choices during accumulated money presentation in patients with pathological gambling^34^
	*Economic decision-making task:* no difference between patients with and without ICD during decision-making phase^37^
	*Visual sentence verification task:* no difference between patients with and without previous ICD^32^
Beta	No relationships found in rest, during economic decision-making task, during a stop signal task, or during a visual sentence verification task^29,32,34,36^
Low gamma	No relationship found during economic decision-making task^34^
Gamma	*Stop signal task:* decrease in power and subthalamic-cortical coherence during successful inhibition in patients without ICD but not in patients with ICD^29^
Other (1–35 Hz)	*Go/NoGo task:* QUIP-RS was negatively correlated with positive ERP deflections during stimulus evaluation and with negative ERP deflections during feedback evaluation (i.e., decreased responses with higher QUIP-RS)^31^
*Depressive symptoms*	
Theta	*Resting-state:* lower in the group with depressive symptoms^41^
	*Resting-state:* negatively correlated with HDRS-17 scores^41^
Alpha	*Resting-state*: higher in the group with depressive symptoms^41^
	*Resting-state*: positive correlation with HDRS-17 scores^41^
	*Emotional processing task:* attenuated ERD in response to positive stimuli compared to controls^38^
	*Emotional processing task:* ERD-index correlated positively with BDI scores at time of recording and 3 months postoperatively^38^
	*Emotional processing task:* negative correlation between ERD after negative stimuli and BDI 3 months postoperatively^38^
	*Emotional processing task:* increased ERD in response to negative stimuli compared to controls^39^
Other (5–20 Hz)	Positive correlation with BDI scores in the ventromedial STN and near the ventromedial border of the STN^40^
*Hypomania*	
Theta	Higher in hemispheres with HIC than in hemispheres without^42^
	Correlation between depth of HIC and depth of maximum power^42^
Alpha	No correlation with depth of HIC and depth of maximum power^42^
Beta	No difference in maximum power between sides with and without HICs^42^
	No correlation with depth of HIC and depth of maximum power^42^
*Cognition*	
Theta-alpha	Positive correlation with phonemic fluency performance^44^
Beta	No correlation with phonemic fluency performance^43^
Gamma	Positive correlation with semantic fluency score during first 15 s for all contacts^43^
	Positive correlation with average total score and average number of switching between categories for all left contacts^43^
	No correlation with phonemic fluency performance^43^
Other (5–20 Hz)	Negative correlation with FAB scores in the ventromedial STN^40^
	No correlation with ACE scores^40^
*Sleep-wake disorders*	
Theta	Negative correlation between low-to-high (theta over sum of beta and low gamma) power ratio and both arousal and sleep fragmentation, for both REM and NREM sleep^47^
Beta	Increased synchronization during REM sleep movements^45^
	Higher basal ganglia-EMG connectivity during REM sleep in patients with RBD compared to those without^46^
	Positive correlation between basal ganglia-EMG connectivity and both RBD-SQ score and video-based RBD severity^46^
	Positive correlation with arousal and sleep fragmentation in NREM sleep^47^
	Negative correlation between low-to-high (theta over sum of beta and low gamma) power ratio and both arousal and sleep fragmentation, for both REM and NREM sleep^47^
	No correlation with the RBD-SQ scores during REM sleep^46^
Low gamma	Negative correlation between low-to-high (theta over sum of beta and low gamma) power ratio and both arousal and sleep fragmentation, for both REM and NREM sleep^47^
*Lower urinary tract symptoms*	
Beta	*During voiding:* no correlation with ICIQ scores^48^
**Globus pallidus interna**	
*Impulse control disorders*	
Other (1–35 Hz)	*Go/NoGo task*: QUIP-RS was positively correlated with positive ERP deflections during stimulus evaluation and with negative ERP deflections during feedback evaluation (i.e., increased responses with higher QUIP-RS)^31^
*Sleep-wake disorders*	
Beta	Increased synchronization during REM sleep movements^49^
	Positive correlation with RBD-SQ during NREM sleep^46,50^
	Higher basal ganglia-EMG connectivity during REM sleep in patients with RBD compared to those without^46^
	Positive correlation between basal ganglia-EMG connectivity and both RBD-SQ score and video-based RBD severity^46^
	Positive correlation with PSQI scores during NREM sleep^50^
	No correlation with the RBD-SQ scores during REM sleep^50^
HFO	Increased during both REM sleep movements and movements during wake^49^
*Lower urinary tract symptoms*	
Beta	*During voiding:* correlation with urgency and incontinence but not voiding score of the ICIQ^48^

ACE: Addenbrooke's Cognitive Examination; ARI: arousal index; BDI: Beck Depression Inventory; BIS-11: 11-item Barratt Impulsiveness Scale; ERD: event-related desynchronization; ERP: event-related potential; ERS: event-related synchronization; FAB: Frontal Assessment Battery; HIC: hypomania inducting contact; HDRS-17: 17-item Hamilton Depression Rating Scale; ICD: impulse control disorders; ICIQ: International Consultation on Incontinence Questionnaire; NREM sleep: non-REM sleep; PSQI: Pittsburgh Sleep Quality Index; QUIP: Questionnaire for Impulsive-Compulsive Disorders in Parkinson's Disease; QUIP-RS: Questionnaire for Impulsive-Compulsive Disorders in Parkinson's Disease – Rating Scale; RBD: REM sleep behavior disorder; RBD-SQ: RBD Screening Questionnaire; REM sleep: rapid eye movement sleep; SFI: sleep fragmentation index.

The effect of motor severity as confounder for NMS severity was controlled for by six studies, either by testing for group differences in scores on the Unified Parkinson's Disease Rating Scale (UPDRS) part III^34,36,37,42^ or by both testing for group differences in UPDRS parts III and IV and including UPDRS-III scores in the statistical model used.^35^ No group differences were found and the UPDRS-III scores did not contribute significantly to the model. Two other studies performed correlation analyses on both their non-motor outcomes and the UPDRS-III scores of which the latter was not statistically significant.^41,50^

Overall, the frequency bands were defined similarly across studies, with the most significant variations observed in the gamma band. The definitions of frequency bands from each study and their classification within this review are summarized in Supplementary Table S2. In one study, the 5–13 Hz frequency band was referred to as the “low-frequency” band but which will be referred to as “theta-alpha” in this manuscript as to align with other studies.^32^ Another study did not predefine frequency bands but identified clusters of significant correlation with its outcomes on a frequency-spatial location plot.^40^

### Impulse control disorders

Studies on ICD measured LFP signals either during rest (n = 2) or while performing a behavioral task (n = 6) such as an economic decision-making task. In studies using resting-state STN-LFPs, patients with ICD, compared to those without, exhibited a greater relative increase in theta-alpha (4–10 Hz), but not beta (12–30 Hz) power, following administration of dopaminergic medication.^36^ Additionally, patients with ICD demonstrated increased subthalamic-cortical coherence within the theta (4–7.5 Hz) band compared to patients without ICD. This increased coherence was primarily observed between the STN and the premotor and dorsolateral prefrontal cortical areas. Alpha band (8–13 Hz) power was not shown to correlate with ICD severity as assessed by the Questionnaire for Impulsive-Compulsive Disorders in Parkinson's disease–Rating Scale (QUIP-RS) but increased with higher impulsiveness, as measured by the 11-item Barratt Impulsiveness Scale (BIS-11).^35^

#### Reward and loss processing

One task investigating reward and loss processing is an economic decision-making paradigm in which participants can earn or lose virtual money by choosing between low-risk and high-risk options. After each choice, the outcome of the previous decision is presented. In one study using this task, the spectral content of the STN during the outcome presentation phase differed between patients with and without pathological gambling.^34^ This difference was primarily attributed to higher beta-range activity in patients with pathological gambling, with the most pronounced difference observed around 19 Hz. Low frequency (LF; 1–12 Hz) power during outcome presentation was lower preceding low-risk choices compared to high-risk choices in patients with pathological gambling, but not in those without. When using the same task but focusing on the decision-making phase, no difference was found in LF power in the STN between patients with and without pathological gambling.^37^

A monetary incentive task was used in another study, in which participants had to respond with correct keyboard responses in order to win or avoid loss of virtual money.^33^ During this task, delta band (2–4 Hz) power in the STN during loss outcome presentation was negatively correlated with scores on the Questionnaire for Impulsive-Compulsive Disorders in Parkinson's disease (QUIP). Conversely, theta band power (4–8 Hz) was positively correlated with QUIP scores during reward outcome. With regards to these results it should be noted that the QUIP, unlike the QUIP-RS, was not developed to assess the severity of ICD.^51^

In reward trials versus avoid-loss trials of a Go/NoGo task, scores on the QUIP-RS were negatively correlated to the 1–35 Hz ERP of the STN.^31^ The ERP increase and decrease during stimulus evaluation and feedback evaluation, respectively, were attenuated in patients with high compared to low QUIP-RS scores. The reverse was seen in the GPi, with increased responses during stimulus and feedback processing for patients with higher QUIP-RS scores.^31^

#### Response inhibition

Successful response inhibition during a stop-signal task was associated with a decrease in both the power and cortico-subthalamic coherence of the STN gamma band (55–75 Hz) in patients without ICD, but not in those with ICD.^29^ This relationship was mostly limited to the ON medication state and was not present for beta (12–30 Hz) and theta (4–7 Hz) power.

#### Moral evaluation

One study used a visual sentence verification task during which patients were asked whether they agreed with presented neutral, moral conflictual, or moral nonconflictual sentences.^32^ Comparing patients with and without previous ICD, no differences were found in STN theta-alpha (5–13 Hz) or beta (14–30 Hz) power during decision-making. However, it was not clear according to which criteria the ICD was diagnosed and whether the disorder was still present when the study took place.

### Depressive symptoms

All included studies on depressive symptoms recorded LFPs from the STN. In one study, the resting-state power in the theta band (4–7 Hz) was shown to be lower in the group with depressive symptoms compared to those without.^41^ The power in the theta band was also negatively correlated with depression severity as measured by the 17-item Hamilton Depression Rating Scale (HDRS-17). Conversely, power in the alpha band (7–13 Hz) was higher in the group with depressive symptoms and was positively correlated with HDRS-17 scores.^41^ This seems to be in contrast with results showing that 5–20 Hz frequency band power (i.e., high theta, alpha and low beta) from the ventromedial STN correlated negatively with scores on the Beck Depression Inventory (BDI).^40^

In two other studies, patients performed an emotional processing task during which pleasant, neutral, and unpleasant pictures were presented.^38,39^ When viewing pleasant pictures, patients with mild to moderate depressive symptoms (BDI ≥ 9) had an attenuated decrease in alpha power compared to patients without depressive symptoms (BDI < 9).^38^ The inverse of this relationship was seen during the viewing of unpleasant pictures, with more alpha power decrease for depressed patients (BDI 17–24) compared to non-depressed patients (BDI <17).^39^ Furthermore, the difference between the decrease after viewing pleasant and unpleasant pictures, or the alpha event-related desynchronization index (alpha-ERD index), correlated positively with the BDI scores both at time of recording and 3 months postoperatively.^38^ A negative correlation was found between the alpha power decrease after unpleasant stimuli and the BDI scores 3 months postoperatively.^38^ In other words, more decrease in alpha power after unpleasant stimuli was associated with higher BDI scores after 3 months.

### Hypomania

When comparing electrodes with and without a hypomania-inducing contact (HIC) in one study, theta (4–7 Hz) but not beta (13–35 Hz) power in the STN was higher in hemispheres with a HIC than in those without.^42^ Additionally, a more ventral position of HICs was associated with a more ventral position of the maximum theta power. This correlation was not found for the maximum alpha (7–10 Hz) and beta power. Gamma (40–60 Hz) power was also reported to have been calculated but no results were reported regarding this frequency band.

### Cognitive dysfunction

Two studies have shown that phonemic verbal fluency (VF) performance correlates positively with theta-alpha (5–15 Hz) but not beta or gamma (30–95 Hz) band power recorded in the STN.^30,43^ Performance of semantic fluency correlated positively with gamma power during the first 15 seconds.^43^ Gamma power within the left STN was also positively related to the average total score and the average number of switching between semantic categories. Beta activity was not related to any semantic fluency measure.

Another study made use of intraoperative LFP signals recorded throughout the implantation trajectory.^40^ Within the ventromedial STN and near the ventromedial border of the STN, a cluster of significant positive correlation was found between 3–25 Hz band power and Frontal Assessment Battery (FAB) scores. No statistically significant correlations were found for Addenbrooke's Cognitive Examination (ACE) scores.

### Sleep-wake disorders

Two studies on RBD have shown that beta power in the GPi and STN decreased during awake movements but increased preceding and during REM sleep movements.^45,49^ In contrast, high frequency oscillations (HFO; 150–350 Hz) in the GPi increased during both REM sleep movements and movements during wake.^49^ Interestingly, other studies found that beta power during NREM but not REM sleep correlated positively with RBD Screening Questionnaire (RBD-SQ) scores, although the former relationship was not tested for the STN.^46,50^ It is important to note that the RBD-SQ was not designed to assess symptom severity and has demonstrated low sensitivity in a cohort of *de novo* PD patients.^52,53^ Patients with high scores on this questionnaire often experience sleep-wake disorders other than RBD.^52,54^ Furthermore, basal ganglia-EMG connectivity of both STN and GPi beta power during REM sleep was higher in patients with RBD compared to those without, and correlated with both RBD-SQ and video-based RBD scores.^46^

Worse sleep quality, measured using the Pittsburgh Sleep Quality Index (PSQI), was related to higher GPi beta band power during NREM sleep but not REM sleep.^50^ This correlation was stronger for the high beta band (20–30 Hz) alone. In addition, the degree of sleep fragmentation, measured using a polysomnography-based sleep fragmentation index (SFI), was shown to correlate positively with both beta and low gamma power during NREM sleep.^47^ The degree of arousal during sleep, measured using an EEG-based arousal index (ARI), was correlated positively with beta power and negatively with theta power during NREM sleep.^47^ Negative correlations were found for the STN between arousal and sleep fragmentation for the ratio of low (theta) to high (beta and low gamma) power during both REM and NREM sleep.^47^ Less theta and/or more beta and low gamma power was thus associated with more sleep fragmentation and higher arousal during all sleep stages.

### Lower urinary tract symptoms

The relationship between LUTS and beta activity was assessed in one study which performed LFP measurements in both the STN and GPi during voiding.^48^ This study found that the beta band (12–25 Hz) power in the GPi correlated positively to the urgency and incontinence but not the voiding score of the International Consultation on Incontinence Questionnaire (ICIQ) on LUTS. No correlation was found between STN beta band power and ICIQ scores.

## Discussion

**Figure 2.**
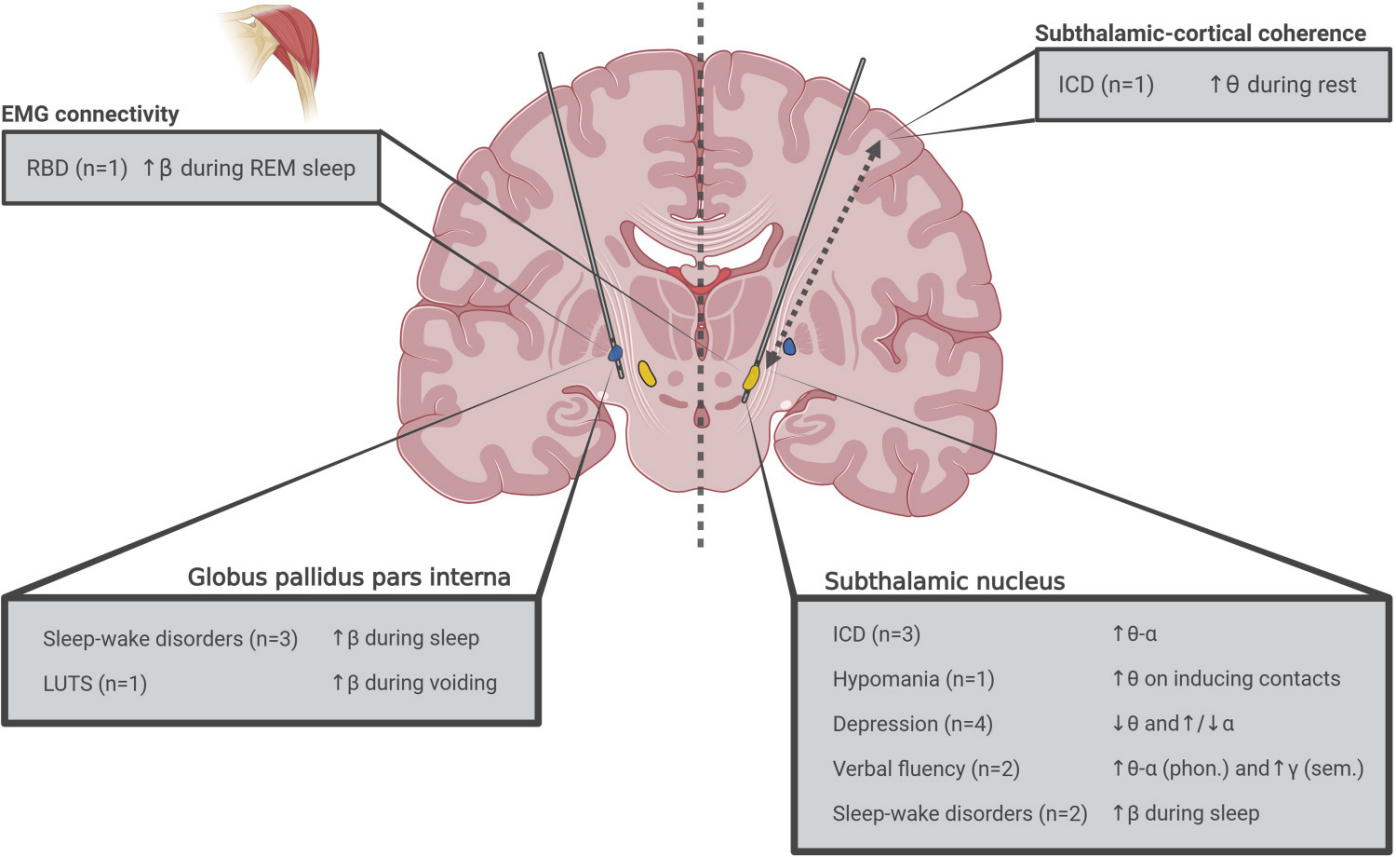
Overview of potential physiomarkers for non-motor symptoms of Parkinson disease as identified by this review. Behind each non-motor symptom, the number of studies supporting each finding is stated. EMG: electromyography; ICD: impulse control disorders; LUTS: lower urinary tract symptoms; phon: phonemic; REM sleep: rapid eye movement sleep; RBD: REM sleep behavior disorder; sem.: semantic; α: alpha activity; β: beta activity; γ: gamma activity; θ: theta activity.

This systematic literature review examined current data linking basal ganglia LFPs to NMS in PD. Despite an overall high risk of bias and limited applicability of findings, several conclusions can be drawn. First, STN theta and alpha bands have been most frequently implicated in neuropsychiatric and cognitive symptoms of PD. For ICD and hypomania, increased theta power seems to be the most promising physiomarker. Alpha and theta power also emerge as candidate physiomarkers for depressive symptoms, although the direction of this relationship and relevant modulating factors remain unclear. Increased theta-alpha and gamma power have shown potential as indicators of phonemic and semantic fluency, respectively. The presence and severity of sleep-wake disorders and urinary dysfunction are most commonly associated with increased beta power in both STN and GPi. A visual summary of these potential physiomarkers for NMS is provided in Figure 2.

### Impulse control disorders

ICD is primarily associated with increased theta band activity in the STN and appears unrelated to alpha band power. However, an indirect relationship cannot be excluded as patients with ICD exhibit higher trait impulsivity—related to alpha band power—compared to those without ICD.^55^ Studies using behavioral tasks suggest that neurophysiological differences between PD patients with and without ICD are most evident during the processing of loss and reward outcomes, potentially influenced by the level of risk involved. While these findings are insightful, translating them into clinical practice remains challenging.

### Depressive symptoms

Two intraoperative studies reported conflicting findings regarding the directionality of the relation between alpha power and depressive symptoms.^40,41^ This discrepancy may partly be attributed to methodological differences, as one study conducted recordings under general anesthesia, while the other involved awake participants. Because the other studies on depressive symptoms made use of an emotional processing task and used different definitions for their depressive and non-depressive groups, their results remain difficult to interpret.^38,39^

### Hypomania

According to one study, stimulation of more ventral contacts in the STN, associated with higher theta power, may increase the risk of stimulation-induced hypomania.^42^ This aligns with findings from other studies reporting hypomania when stimulating ventral contacts located in the limbic part of the STN.^56–58^ However, the relationship between STN theta power and the severity of hypomania has not yet been investigated.

### Cognitive dysfunction

At the group level, postoperative worsening of verbal fluency has consistently been reported in DBS patients compared to controls.^9,11,59^ Although still not fully understood, this effect is likely driven primarily by a surgical micro-lesion effect, with only limited contribution from the stimulation itself.^60–62^ In this review, we identified that phonemic and semantic fluency performance measures were positively correlated with STN theta-alpha and gamma band activity, respectively.^30,43^ This distinction suggests differing underlying mechanisms for phonemic and semantic fluency, consistent with studies that demonstrate distinct neuroanatomical and neuropathological correlates for these two types of verbal fluency.^63,64^

### Sleep-wake disorders

While it has been established that movements during wakefulness are associated with beta desynchronization,^65^ pathological movements caused by RBD appear to be linked to STN and GPi beta synchronization.^45,46,49^ This aberrant beta behavior supports the idea that movement signaling bypasses the basal ganglia during REM sleep.^66,67^ Consequently, beta power may serve as a useful physiomarker for detecting and monitoring RBD in PD.

While beta activity during REM sleep may correlate with pathological movements, increased beta activity during NREM sleep seems to be related to poorer sleep quality and increased sleep fragmentation.^47,50^ Given that STN-DBS is known to reduce beta power, this reduction may contribute to the improvements in sleep quality and duration observed following STN-DBS.^68–71^ As such, beta activity during NREM sleep could potentially serve as a physiomarker for sleep quality and sleep fragmentation.

### Lower urinary tract symptoms

The effect of DBS on urinary symptoms in PD remains unclear. Limited evidence suggests that bladder function improves after STN-DBS but not GPi-DBS.^10,72–74^ However, a small study reported that beta activity in the GPi, but not the STN, was associated with the severity of LUTS.^48^ To better understand the impact of DBS on urinary symptoms and the neurophysiological underpinnings hereof, future studies should consider the effects of dopaminergic drugs, which are often reduced following STN-DBS but not GPi-DBS. Additionally, basal ganglia LFPs should be measured during urinary phases beyond voluntary voiding, such as during storage testing.^75^

### Limitations

This systematic review aimed to address the full spectrum of NMS in PD for which the search strategy was aimed to be as comprehensive and inclusive as possible. However, it remains possible that studies addressing symptoms not encompassed within broader NMS categories were overlooked. In addition, no specific attention was given to a possible confounding effect of motor symptoms in the results. In recent years, several non-motor subtypes of PD have been proposed which not only differ in NMS but also in motor symptom profile and severity.^76,77^ While some of the included studies have controlled for the degree of motor symptoms, this was mostly limited to group comparisons of UPDRS-III scores and most studies did not perform any correction. It could therefore be possible that some of the found relationships between LFP data and NMS are caused by differences in motor symptoms rather than differences in NMS.

A further limitation is the lack of distinction between primary and secondary outcomes in the included studies, which was not accounted for in the risk of bias assessment. Additionally, NMS were not differentiated based on whether they are intrinsic to PD or occur as adverse effects of treatment (e.g., medication-induced ICD or stimulation-induced hypomania). This distinction was not made due to the anticipated small number of studies but it is an important consideration, as the etiology of symptoms significantly influences their treatment and prevention.

### Future research

All studies identified in our review were conducted in hospital settings in defined ON or OFF medication states, and most studies did not assess momentary symptom severity at the time of LFP recordings. However, measurements are likely to be more relevant when performed in naturalistic, unconstrained at-home environments. Such research has become feasible with the introduction of commercially available bidirectional neurostimulators capable of continuously and passively recording LFPs.^78^ This experimental approach provides a unique opportunity to capture the natural fluctuations of non-motor symptoms and could be combined with concurrent assessment of symptom severity, potentially through the use of ecological momentary assessments (EMAs). EMAs involve brief symptom questionnaires completed at several (semi-)random moments throughout the day over a period of days to weeks. These assessments have already been shown to be both feasible and valuable for monitoring motor and non-motor fluctuations in PD.^79,80^

This review highlights that theta-alpha activity is associated with a range of emotional and cognitive neuropsychiatric symptoms. Consistent with this, clinical associations have been observed between depression, ICD, and hypomania in PD.^81–83^ Similarly, beta power has been implicated in both sleep-wake disorders and LUTS, and it is a known physiomarker of poorer motor performance in PD.^18,84^ This raises the question of whether theta-alpha and beta power are shared physiomarkers across these symptom groups, or whether they can be separated. The latter option could allow for more tailored adaptive stimulation algorithms targeting specific symptoms. Recent studies have also shown that theta-frequency stimulation increases theta power on the dorsolateral prefrontal cortex^85^ and also yields positive effects on verbal frequency, working memory, and cognitive control and the processing bias toward negative stimuli as seen in depressed patients.^86–89^ This suggests a promising avenue for developing targeted, principled neuromodulation strategies for these symptoms. For example, theta-alpha-informed aDBS algorithms delivering theta-frequency stimulation could be designed. However, this approach faces a practical challenge: recording and stimulating at the same frequency is not possible with the current DBS hardware.

In addition to band power, differences in other LFP characteristics could contribute to further disentangle symptoms. For instance, subthalamic-cortical coherence and burst dynamics in the beta band have already been shown to reflect medication state and PD motor subtype.^71,90–92^ Furthermore, recent studies have linked medication state and motor severity to the aperiodic (non-oscillatory) component of the subthalamic LFP signal.^93,94^ Additionally, data-driven approaches that utilize raw LFP signals could potentially yield more accurate, albeit more complex, physiomarkers for the intricate neuronal disruptions underlying NMS. Lastly, only one study included in this systematic review has looked at the association between LFP measure amplitude and spatial location of electrode contacts while none has assessed the temporal behavior of potential physiomarkers other than in relation to behavioral paradigms.^42^ Spatial (e.g., location within the STN) and temporal (e.g., night versus day) differences could further aid in distinguishing one symptom from another. High-resolution imaging combined with neurophysiological source localization techniques could provide valuable insights into the neurophysiological basis and spatial separation of different NMS.

For the translation of this knowledge into clinical practice, it will be essential to differentiate between PD-specific NMS and stimulation-induced symptoms, as each likely requires a distinct approach. More importantly, future studies should shift their focus from group-level differences to individual-specific physiomarkers. This will allow DBS treatment to be more precisely tailored to each patient, ultimately improving quality of life.

## Conclusion

With the current knowledge, basal ganglia theta and alpha activity seem to be the most promising physiomarkers of several neuropsychiatric disorders, whereas beta activity shows the most promise in identifying sleep-wake disorders and urinary dysfunction in patients with PD. The upcoming challenges within this field will be to discover and validate physiomarkers in a naturalistic and within-subject setting, and to develop responsive algorithms aimed at improving these symptoms. Eventually, these developments could lead to novel symptom- and patient-specific stimulation paradigms which will further improve care and outcomes for PD.

## Supplemental Material

sj-docx-1-pkn-10.1177_1877718X261459224 - Supplemental material for Basal ganglia neurophysiological markers of non-motor symptoms in Parkinson's disease: A systematic review Supplemental material, sj-docx-1-pkn-10.1177_1877718X261459224 for Basal ganglia neurophysiological markers of non-motor symptoms in Parkinson's disease: A systematic review by Bart J Keulen, Martijn Beudel, Arjan Malekzadeh, Rob MA de Bie and Bart EKS Swinnen in Journal of Parkinson's Disease
