# Supplementary material for: Basal ganglia neurophysiological markers of non-motor symptoms in Parkinson's disease: A systematic review
Source: J Parkinsons Dis. 2026 Jun 23;16(5):826–41. doi: 10.1177/1877718X261459224 (PMC13435217; doi:10.1177/1877718X261459224)
Supplement: Supplementary file 1 [file sj-docx-1-pkn-10.1177_1877718X261459224.docx]

Supplementary Materials

Basal ganglia physiomarkers of non-motor symptoms in Parkinson’s disease: a systematic review

B.J. Keulen, MSc^1^, M. Beudel, MD PhD^1^, A. Malekzadeh, PhD^2^, R.M.A. de Bie, MD PhD^1^, B.E.K.S. Swinnen, MD PhD^1,3^

*^1^* Amsterdam University Medical Center, Department of Neurology, Amsterdam Neuroscience, University of Amsterdam, Amsterdam, The Netherlands

*^2^* Amsterdam University Medical Center, Medical Library, University of Amsterdam, Amsterdam, The Netherlands

^3^ University Hospitals Leuven, Department of Neurology, Leuven, Belgium

**Contact information**

Bart J. Keulen, MSc

Amsterdam UMC
Dept. of Neurology, room H2-235
Meibergdreef 9
1105 AZ Amsterdam
The Netherlands

+31 (0)20-5663446
[b.j.keulen@amsterdamumc.nl](mailto:b.j.keulen@amsterdamumc.nl)

**Contents:**

- Supplementary Table S1. Prediction model Risk Of Bias ASsessment Tool (PROBAST) assessment of risk of bias and concern regarding applicability for each included study.
- Supplementary Table S2. Overview of the definitions of frequency bands as used in the included articles
- Search strategy: MEDLINE (Ovid)
- Search strategy: Embase (Ovid)
- Search strategy: Scopus
- Search strategy: Web of Science

**Supplementary Table S1** Prediction model Risk Of Bias ASsessment Tool (PROBAST) assessment of risk of bias and concern regarding applicability for each included study

| First Author | Year | Risk of bias | | | | | Concern regarding applicability | | | |
| --- | --- | --- | --- | --- | --- | --- | --- | --- | --- | --- |
|  |  | **Participants** | **Predictors** | **Outcome** | **Analysis** | **Overall** | **Participants** | **Predictors** | **Outcome** | **Overall** |
| Alegre^1^ | 2013 | Low | Low | High | High | High | Low | Low | Low | Low |
| Anzak^2^ | 2011 | Low | Low | Unclear | Unclear | Unclear | Low | Low | Unclear | Unclear |
| Chen^3^ | 2021 | Low | Low | Low | Unclear | Unclear | Low | Low | Low | Low |
| Eisinger^4^ | 2022 | Low | Low | Low | Low | Low | Low | Unclear | Low | Unclear |
| Fumagalli^5^ | 2011 | Low | Low | High | Low | High | Low | Unclear | High | High |
| Hackius^6^ | 2016 | Low | Low | Low | High | High | Low | Unclear | Low | Unclear |
| Huebl^7^ | 2011 | Low | Low | Low | Low | Low | Low | Unclear | Low | Unclear |
| Huebl^8^ | 2014 | Low | Low | Low | High | High | Low | Unclear | Low | Unclear |
| Manssuer^9^ | 2021 | Low | Low | Low | High | High | Low | Unclear | High | High |
| Mazzoni^10^ | 2018 | High | Low | Low | Unclear | High | Low | Unclear | Low | Unclear |
| Rappel^11^ | 2020 | Low | Low | Low | Unclear | Unclear | Low | Low | Low | Low |
| Ricciardi^12^ | 2021 | Low | Low | Low | Low | Low | Low | Low | Low | Low |
| Rodriquez-Oroz^13^ | 2010 | Low | Low | Low | Low | Low | Low | Low | Low | Low |
| Rosa^14^ | 2013 | Low | Low | Low | Low | Low | Low | Unclear | Low | Low |
| Roy^15^ | 2018 | Low | Low | Low | Unclear | Unclear | Low | Low | Low | Low |
| Sun^16^ | 2021 | Low | Low | Low | Low | Low | Low | Low | Low | Low |
| Verma^17^ | 2022 | Low | Low | Low | High | Unclear | Low | Unclear | Low | Unclear |
| Wojtecki^18^ | 2016 | Low | Low | Low | Unclear | Unclear | Low | Unclear | Low | Unclear |
| Yin^19^ | 2023 | Low | Low | Low | Low | Low | Low | Low | High | High |
| Yin^20^ | 2024 | Low | Low | Low | Low | Low | Low | Low | Low | Low |
| Zhang^21^ | 2024 | Low | Low | Unclear | Low | Unclear | Low | Low | Unclear | Unclear |

**Supplementary Table S2** Overview of the definitions of frequency bands as used in the included articles

| **Frequency band** | | | | | |
| --- | --- | --- | --- | --- | --- |
| Delta | 1-3 Hz ^21^ | 2-4 Hz^9^ |  |  |  |
| Theta | 4-7 Hz^3, 16^ | 4-7.5 Hz^13^ | 4-8 Hz^9, 20^ |  |  |
| Theta-alpha | 4-10 Hz^13^ | 5-13 Hz^5^ | 5-15 Hz^18^ |  |  |
| Alpha | 7-10 Hz^3^ | 7-13 Hz^16^ | 8-12 Hz^7, 8^ | 8-13 Hz^12^ | 8-14 Hz^21^ |
| Low frequency | 1-12 Hz^10^ | 2-12 Hz^14^ |  |  |  |
| Beta | 12-25 Hz^15^ | 12-30 Hz^10, 13^ | 13-30 Hz^2, 19, 20^ | 13-35 Hz^3, 6, 17^ | 14-30 Hz^5^ |
| Low gamma | 30-50 Hz^10^ | 30-60 Hz^20^ | 31-60 Hz^21^ |  |  |
| Gamma | 30-95 Hz^2^ | 30-100 Hz^18^ | 55-75 Hz^1^ |  |  |
| HFO | 150-350 Hz^17^ |  |  |  |  |
| Other | 1-35 Hz^4^ | 3-25 Hz^11^* | 5-20 Hz^11^* |  |  |

HFO = high frequency oscillations. *These frequency bands were not predefined but identified using a cluster-based analysis. In the original article, they were referred to as wide delta-low beta (3-25 Hz) and wide theta-alpha and low beta (5-20 Hz).

**Search strategy: MEDLINE (Ovid)**

| Database(s): Ovid MEDLINE(R) ALL 1946 to December 07, 2023 | |
| --- | --- |
| # | Searches |
| 1 | exp Parkinson Disease/ |
| 2 | (paralys* adj3 agitan*).ti,ab,kf. |
| 3 | (shaking adj3 palsy).ti,ab,kf. |
| 4 | parkinson*.ti,ab,kf. |
| 5 | or/1-4 |
| 6 | (LFP or (local* adj2 field* adj2 potential*)).ti,ab,kf. |
| 7 | (local* adj2 synaptic* adj2 potential*).ti,ab,kf. |
| 8 | ((beta or alpha or theta or gamma or delta) adj3 oscillat*).ti,ab,kf. |
| 9 | ((Low* or high*) adj2 frequenc* adj3 Oscillat*).ti,ab,kf. |
| 10 | or/6-9 |
| 11 | exp Cognition Disorders/ or exp Mood Disorders/ or exp Sleep Wake Disorders/ or exp Sensation Disorders/ or exp Somatosensory Disorders/ or Autonomic Nervous System Diseases/ or exp Vision Disorders/ or exp Speech Disorders/ or exp Anxiety/ or exp Apathy/ or exp Delusions/ or exp Hallucinations/ or exp Urinary Incontinence/ or exp Gastrointestinal Diseases/ or exp Executive Function/ or exp Affective Symptoms/ or exp "Disruptive, Impulse Control, and Conduct Disorders"/ or exp Reward/ or exp REM Sleep Behavior Disorder/ or exp Decision Making/ or exp Mental Disorders/ or exp Temperament/ |
| 12 | ((cognit* or visual or speech or REM or behavio?r* or mental* or sleep* or urine or intestin* or executive or mood or sensory or autonomic or affective or emotion* or "impulse control" or conduct* or impulsiv* or compulsiv* or temperament) adj3 (disabilit* or symptom* or disorder* or defect* or function* or deficienc* or impairment* or problem* or dysfunct* or dysregulat* or abnormal*)).ti,ab,kf. |
| 13 | ((non or "not") adj3 (motor* or physiomarker*) adj3 (symptom* or outcome*)).ti,ab,kf. |
| 14 | (incontinence or constipat* or insomnia* or depress* or dementia or hallucination* or novelty or saliency or psycho* or (decision* adj3 making) or reward*).ti,ab,kf. |
| 15 | or/11-14 |
| 16 | 5 and 10 and 15 |
| 17 | ((exp animals/ or exp veterinary medicine/ or animal*.jw.) not exp humans/) or (experiment* model* or animal* or monkey* or sheep or ?ovine or lamb* or goat* or pig* or swine or porcine or pup* or dog* or canine or bitch* or beagle* or feline or rodent* or rabbit* or rat or rats or mouse or murine or mice).ti,kf. |
| 18 | 16 not 17 |

**Search strategy: Embase (Ovid)**

| Database(s): Embase 1974 to 2023 December 07 | |
| --- | --- |
| # | Searches |
| 1 | exp Parkinson disease/ |
| 2 | (paralys* adj3 agitan*).ti,ab,kf. |
| 3 | (shaking adj3 palsy).ti,ab,kf. |
| 4 | parkinson*.ti,ab,kf. |
| 5 | or/1-4 |
| 6 | exp local field potential/ |
| 7 | (LFP or (local* adj2 field* adj2 potential*)).ti,ab,kf. |
| 8 | (local* adj2 synaptic* adj2 potential*).ti,ab,kf. |
| 9 | ((beta or alpha or theta or gamma or delta) adj3 oscillat*).ti,ab,kf. |
| 10 | ((Low* or high*) adj2 frequenc* adj3 Oscillat*).ti,ab,kf. |
| 11 | or/6-10 |
| 12 | exp cognitive defect/ or exp mood disorder/ or exp sleep disorder/ or exp sensory dysfunction/ or exp autonomic dysfunction/ or exp visual disorder/ or exp speech disorder/ or exp anxiety/ or exp apathy/ or exp delusion/ or exp urine incontinence/ or exp intestine function disorder/ or executive function/ or exp emotional disorder/ or exp impulse control disorder/ or exp reward/ or exp rapid eye movement sleep behavior disorder/ or exp decision making/ or exp psychosis/ or exp behavior disorder/ or exp temperament/ |
| 13 | ((cognit* or visual or speech or REM or behavio?r* or mental* or sleep* or urine or intestin* or executive or mood or sensory or autonomic or affective or emotion* or "impulse control" or conduct* or impulsiv* or compulsiv* or temperament) adj3 (disabilit* or symptom* or disorder* or defect* or function* or deficienc* or impairment* or problem* or dysfunct* or dysregulat* or abnormal*)).ti,ab,kf. |
| 14 | ((non or "not") adj3 (motor* or physiomarker*) adj3 (symptom* or outcome*)).ti,ab,kf. |
| 15 | (incontinenc* or constipat* or insomnia* or depress* or dementia or hallucination* or novelty or saliency or psycho* or (decision* adj3 making) or reward*).ti,ab,kf. |
| 16 | or/12-15 |
| 17 | 5 and 11 and 16 |
| 18 | ((exp animals/ or exp veterinary medicine/ or animal*.jw.) not exp humans/) or (experiment* model* or animal* or monkey* or sheep or ?ovine or lamb* or goat* or pig* or swine or porcine or pup* or dog* or canine or bitch* or beagle* or feline or rodent* or rabbit* or rat or rats or mouse or murine or mice).ti,kf. |
| 19 | 17 not 18 |

**Search strategy: Scopus**

Scopus

TITLE-ABS-KEY ( ( parkinson* ) OR ( paralys* W/3 agitan* ) OR ( shaking W/3 palsy ) ) AND TITLE-ABS-KEY ( ( ( lfp OR ( local* W/2 field* W/2 potential* ) ) ) OR ( ( beta OR alpha OR theta OR gamma OR delta ) W/3 oscillat* ) OR ( ( low* OR high* ) W/2 frequenc* W/3 oscillat* ) ) AND TITLE-ABS-KEY-AUTH ( ( ( cognit* OR visual OR speech OR rem OR behavior* OR sleep OR urine OR intestin* OR executive OR mood OR sensory OR autonomic OR affective OR emotion* OR "impulse control" OR conduct* OR impulsiv* OR compulsiv* OR temperament ) W/3 ( disabilit* OR symptom* OR disorder* OR defect* OR function* OR deficienc* OR impairment* OR problem* OR dysfunct* OR dysregulat* ) ) OR ( ( non OR "not" ) W/3 ( motor* OR physiomarker* ) W/3 ( symptom* OR outcome* ) ) OR ( incontinenc* OR constipat* OR insomnia* OR depress* OR dementia OR hallucination* OR novelty OR saliency OR psycho* OR ( decision* W/3 making ) OR reward* ) )

**Search strategy: Web of Science**

\# Web of Science Search Strategy (v0.1)

\# Database: Web of Science Core Collection

- WOS.SCI: 1975 to 2023

- WOS.AHCI: 1975 to 2023

- WOS.ESCI: 2005 to 2023

- WOS.SSCI: 1975 to 2023

\# Searches:

1: TS=(((cognit* or visual or speech or REM or behavior* or sleep or urine or intestin* or executive or mood or sensory or autonomic or affective or emotion* or "impulse control" or conduct* or impulsiv* or compulsiv* or temperament) near/3 (disabilit* or symptom* or disorder* or defect* or function* or deficienc* or impairment* or problem* or dysfunct* or dysregulat*)) or ((non or "not") near/3 (motor* or physiomarker*) near/3 (symptom* or outcome*)) or (incontinenc* or constipat* or insomnia* or depress* or dementia or hallucination* or novelty or saliency or psycho* or (decision* near/3 making) or reward*))

2: TS=(((LFP or (local* near/2 field* near/2 potential*)) or ((beta or alpha or theta or gamma or delta) near/3 oscillat*) or ((Low* or high*) near/2 frequenc* near/3 Oscillat*)))

3: TS=(parkinson* or (paralys* near/3 agitan*) or (shaking near/3 palsy))

4: #1 AND #2 AND #3

**References**

1.	Alegre M, Lopez-Azcarate J, Obeso I, et al. The subthalamic nucleus is involved in successful inhibition in the stop-signal task: A local field potential study in Parkinson's disease. *Exp Neurol* 2013; 239: 1-12. DOI: <https://doi.org/10.1016/j.expneurol.2012.08.027>.

2.	Anzak A, Gaynor L, Beigi M, et al. A gamma band specific role of the subthalamic nucleus in switching during verbal fluency tasks in Parkinson's disease. *Exp Neurol* 2011; 232: 136-142. 20110818. DOI: <https://doi.org/10.1016/j.expneurol.2011.07.010>.

3.	Chen Y-C, Wu H-T, Tu P-H, et al. Theta Oscillations at Subthalamic Region Predicts Hypomania State After Deep Brain Stimulation in Parkinson's Disease. *Front Hum Neurosci* 2021; 15. Original Research. DOI: <https://doi.org/10.3389/fnhum.2021.797314>.

4.	Eisinger RS, Cagle JN, Alcantara JD, et al. Distinct Roles of the Human Subthalamic Nucleus and Dorsal Pallidum in Parkinson’s Disease Impulsivity. *Biol Psychiatry* 2022; 91: 370-379. DOI: <https://doi.org/10.1016/j.biopsych.2021.03.002>.

5.	Fumagalli M, Giannicola G, Rosa M, et al. Conflict-dependent dynamic of subthalamic nucleus oscillations during moral decisions. *Soc Neurosci* 2011; 6: 243-256. DOI: <https://doi.org/10.1080/17470919.2010.515148>.

6.	Hackius M, Werth E, Sürücü O, et al. Electrophysiological Evidence for Alternative Motor Networks in REM Sleep Behavior Disorder. *J Neurosci* 2016; 36: 11795-11800. DOI: <https://doi.org/10.1523/jneurosci.2546-16.2016>.

7.	Huebl J, Schoenecker T, Siegert S, et al. Modulation of subthalamic alpha activity to emotional stimuli correlates with depressive symptoms in Parkinson's disease. *Mov Disord* 2011; 26: 477-483. 20110201. DOI: <https://doi.org/10.1002/mds.23515>.

8.	Huebl J, Spitzer B, Brücke C, et al. Oscillatory subthalamic nucleus activity is modulated by dopamine during emotional processing in Parkinson's disease. *Cortex* 2014; 60: 69-81. DOI: <https://doi.org/10.1016/j.cortex.2014.02.019>.

9.	Manssuer L, Wang L, Ding Q, et al. Subthalamic Oscillatory Activity of Reward and Loss Processing Using the Monetary Incentive Delay Task in Parkinson Disease. *Neuromodulation* 2023; 26: 414-423. DOI: <https://doi.org/10.1016/j.neurom.2022.04.033>.

10.	Mazzoni A, Rosa M, Carpaneto J, et al. Subthalamic Neural Activity Patterns Anticipate Economic Risk Decisions in Gambling. *eNeuro* 2018; 5 20180206. DOI: <https://doi.org/10.1523/eneuro.0366-17.2017>.

11.	Rappel P, Grosberg S, Arkadir D, et al. Theta-alpha oscillations characterize emotional subregion in the human ventral subthalamic nucleus. *Mov Disord* 2020; 35: 337-343. 20191123. DOI: <https://doi.org/10.1002/mds.27910>.

12.	Ricciardi L, Fischer P, Mostofi A, et al. Neurophysiological Correlates of Trait Impulsivity in Parkinson's Disease. *Mov Disord* 2021; 36: 2126-2135. 20210513. DOI: <https://doi.org/10.1002/mds.28625>.

13.	Rodriguez-Oroz MC, López-Azcárate J, Garcia-Garcia D, et al. Involvement of the subthalamic nucleus in impulse control disorders associated with Parkinson’s disease. *Brain* 2010; 134: 36-49. DOI: <https://doi.org/10.1093/brain/awq301>.

14.	Rosa M, Fumagalli M, Giannicola G, et al. Pathological gambling in Parkinson's disease: subthalamic oscillations during economics decisions. *Mov Disord* 2013; 28: 1644-1652. 20130402. DOI: <https://doi.org/10.1002/mds.25427>.

15.	Roy HA, Aziz TZ, Fitzgerald JJ, et al. Beta oscillations and urinary voiding in Parkinson disease. *Neurology* 2018; 90: e1530-e1534. 20180323. DOI: <https://doi.org/10.1212/wnl.0000000000005355>.

16.	Sun Y, Wang Z, Hu K, et al. α and θ oscillations in the subthalamic nucleus are potential biomarkers for Parkinson's disease with depressive symptoms. *Parkinsonism Relat Disord* 2021; 90: 98-104. 20210724. DOI: <https://doi.org/10.1016/j.parkreldis.2021.07.023>.

17.	Verma AK, Acosta Lenis SF, Aman JE, et al. Basal ganglia engagement during REM sleep movements in Parkinson’s disease. *npj Parkinsons Dis* 2022; 8: 116. DOI: <https://doi.org/10.1038/s41531-022-00382-z>.

18.	Wojtecki L, Elben S, Vesper J, et al. The rhythm of the executive gate of speech: subthalamic low-frequency oscillations increase during verbal generation. *Eur J Neurosci* 2017; 45: 1200-1211. DOI: <https://doi.org/10.1111/ejn.13429>.

19.	Yin Z, Ma R, An Q, et al. Pathological pallidal beta activity in Parkinson's disease is sustained during sleep and associated with sleep disturbance. *Nat Commun* 2023; 14: 5434. 20230905. DOI: <https://doi.org/10.1038/s41467-023-41128-6>.

20.	Yin Z, Yuan T, Yang A, et al. Contribution of basal ganglia activity to REM sleep disorder in Parkinson's disease. *J Neurol Neurosurg Psychiatry* 2024 20240419. DOI: <https://doi.org/10.1136/jnnp-2023-332014>.

21.	Zhang G, Yu H, Chen Y, et al. Neurophysiological features of STN LFP underlying sleep fragmentation in Parkinson's disease. *J Neurol Neurosurg Psychiatry* 2024 20240509. DOI: <https://doi.org/10.1136/jnnp-2023-331979>.
